# Excess Deaths during Influenza and Coronavirus Disease and Infection-Fatality Rate for Severe Acute Respiratory Syndrome Coronavirus 2, the Netherlands

**DOI:** 10.3201/eid2702.202999

**Published:** 2021-02

**Authors:** Liselotte van Asten, Carel N. Harmsen, Lenny Stoeldraijer, Don Klinkenberg, Anne C. Teirlinck, Marit M.A. de Lange, Adam Meijer, Jan van de Kassteele, Arianne B. van Gageldonk-Lafeber, Susan van den Hof, Wim van der Hoek

**Affiliations:** National Institute for Public Health and the Environment, Bilthoven, the Netherlands (L. van Asten, D. Klinkenberg, A.C. Teirlinck, M.M.A. de Lange, A. Meijer, J. van de Kassteele, A.B. van Gageldonk-Lafeber, S. van den Hof, W. van der Hoek);; Statistics Netherlands, the Hague, the Netherlands (C.N. Harmsen, L. Stoeldraijer)

**Keywords:** surveillance, influenza, influenza viruses, coronavirus disease, COVID-19, severe acute respiratory syndrome coronavirus 2, SARS-CoV-2, coronavirus, viruses, epidemics, excess deaths, mortality, mortality rate, infection-fatality rate, trends, time series, seasonality, respiratory infections, zoonoses, the Netherlands

## Abstract

Since the 2009 influenza pandemic, the Netherlands has used a weekly death monitoring system to estimate deaths in excess of expectations. We present estimates of excess deaths during the ongoing coronavirus disease (COVID-19) epidemic and 10 previous influenza epidemics. Excess deaths per influenza epidemic averaged 4,000. The estimated 9,554 excess deaths (41% in excess) during the COVID-19 epidemic weeks 12–19 of 2020 appeared comparable to the 9,373 excess deaths (18%) during the severe influenza epidemic of 2017–18. However, these deaths occurred in a shorter time, had a higher peak, and were mitigated by nonpharmaceutical control measures. Excess deaths were 1.8-fold higher than reported laboratory-confirmed COVID-19 deaths (5,449). Based on excess deaths and preliminary results from seroepidemiologic studies, we estimated the infection-fatality rate to be 1%. Monitoring of excess deaths is crucial for timely estimates of disease burden for influenza and COVID-19. Our data complement laboratory-confirmed COVID-19 death reports and enable comparisons between epidemics.

Influenza and coronavirus disease (COVID-19) are respiratory illnesses that show a high burden of disease. Comparison of their effect on death rates is critical in light of the discussion, especially early in the COVID-19 pandemic, whether deaths from COVID-19 are comparable to or higher than deaths from influenza. With the yearly influenza season nearing the Northern Hemisphere, the comparison of burden will remain essential because the 2 viruses might continue to affect populations.

The ongoing COVID-19 pandemic and previous seasonal influenza epidemics have led to deaths exceeding the levels that are normally expected in a certain period (i.e., excess deaths) (*1*,*2*). Although the contribution of either COVID-19 or influenza (*3*,*4*) to excess all-cause deaths can only be estimated, this information is pivotal for real-time monitoring of the impact and severity of any epidemic (*5*) by providing timely and inclusive estimates (*6*). In the case of influenza, there is no alternative to estimates because only a fraction of patients with influenza-like illness (ILI) or severe acute respiratory infections are tested for influenza virus infection (*7*). Thus, the number of laboratory-confirmed influenza deaths is not a useful indicator and it is also delayed. COVID-19, which must be reported, is subject to closer monitoring and more extensive laboratory testing than influenza in most countries. However, as with influenza, deaths from laboratory-confirmed cases of COVID-19 are an underestimate of the total number of deaths from this disease.

We provide an estimate of the excess deaths observed during the COVID-19 epidemic in the Netherlands in March–May 2020, in comparison with excess deaths observed during the previous 10 influenza epidemics. In addition, we compared the excess COVID-19 death estimates with reported COVID-19 deaths and provide a timely preliminary estimate of the infection-fatality rate of COVID-19.

## Methods

### Data

To estimate excess deaths during influenza and COVID-19 epidemics and their relationship with reported COVID-19 deaths, we used the following resources. First, weekly number of deaths are monitored at the National Institute for Public Health and the Environment (Bilthoven, the Netherlands [RIVM]) by using death registrations from Statistics Netherlands with a 100% coverage of the country (total 2019 population 17.3 million). The monitoring was implemented in 2009 during the influenza pandemic (*1*,*8*,*9*). RIVM receives data from Statistics Netherlands every Thursday. For our analyses, we used data and results from this system for 2010–2020 (2020 data through week 25, ending June 17). Aggregated weekly numbers (running from Thursday through Wednesday for the most up-to-date reporting) were used (Monday–Sunday definitive numbers available at https://opendata.cbs.nl).

Second, influenza epidemic weeks (2010–2020) are defined and reported weekly and yearly by the national sentinel influenza surveillance system (*7*). In this system, the incidence of medically attended ILI incidence is registered by Nivel Primary Care Database based on its sentinel general practitioner practices (*10*). A subgroup of patients with ILI and other acute respiratory infections is swabbed for laboratory testing. Swab specimens are analyzed for influenza virus, respiratory syncytial virus, rhinovirus, enterovirus, and, since February 2020, severe acute respiratory syndrome coronavirus 2 (SARS-CoV-2). This system covers 0.7%–0.8% of the population of the Netherlands and is nationally representative for age, sex, regional distribution, and population density (*11*). When ILI incidence is above the preset threshold for >2 consecutive weeks (*12*), and when influenza virus is detected in samples from patients who have ILI, an influenza epidemic is declared and reported.

Third, patients with laboratory confirmed COVID-19 diagnosis must be reported to regional public health services and their data are entered into a national database maintained by RIVM (February 2020, https://www.rivm.nl/en/novel-coronavirus-covid-19/current-information; open data at https://data.rivm.nl). Records include date of death, if applicable. Because most persons are not deceased when first reported, record completion requires follow-up of patients. For our study, reports by date of death were aggregated weekly from Thursday through Wednesday to enable comparison with death monitoring.

### Defining Periods of Influenza, COVID-19, and Mixed Epidemics

The 2020 seasonal influenza epidemic was short and mild, running from week 5 through week 7. On Thursday, February 27, 2020, the first new SARS-CoV-2 infection was detected in the Netherlands (https://www.rivm.nl/coronavirus-covid-19/grafieken). This infection was at the end of calendar week 9, or week 10 in our weekly Thursday–Wednesday aggregations: February 27–March 4 (Table 1). The seasonal influenza epidemic appeared to resurface briefly in weeks 10 and 11 (February 27–March 11) and overlapped with the first 2 full weeks for reported COVID-19 patients. Therefore, we analyzed separately the cumulated excess deaths for these 2 weeks of mixed epidemics. Fear of coronavirus infection might have motivated persons who had ILI but who would not otherwise have sought care to visit their physician, causing or heightening an increase in the ILI surveillance data. However, a true resurfacing of influenza could not be ruled out; although not common, resurfacing has occurred (*13*,*14*). In both weeks (weeks 10–11 2020), influenza virus was detected in swabbed ILI patients (40% in week 10). In week 11, SARS-CoV-2 was detected in primary care, although at less than half the level of influenza: 10% vs. 25% of ILI patients (*15*) (but based on low numbers of swabbed patients). We counted excess deaths during the COVID-19 epidemic from week 12 through week 19 (March 12–May 6). By week 20, death levels had returned to expected levels, although COVID-19 death reports persisted at low levels (≈0.07 deaths/100,000 persons; https://www.rivm.nl/coronavirus-covid-19/grafieken).

**Table 1:** Total, excess, and reported COVID-19 deaths during 25 weeks of the COVID-19 epidemic, the Netherlands*

Week†	Week start (Thursday)	Week end (Wednesday)	No. observed all-cause deaths	No. expected all-cause deaths (baseline)	No. excess deaths	% Above expected	No. reported COVID-19 deaths	Ratio (excess vs. reported COVID-19 deaths)
1	2019 Dec 26	2020 Jan 1	2,982	2,972	10	0	NA	NA
2	2020 Jan 2	2020 Jan 8	3,166	2,985	181	6	NA	NA
3	2020 Jan 9	2020 Jan 15	3,342	2,996	346	12	NA	NA
4	2020 Jan 16	2020 Jan 22	2,986	3,004	−18	−1	NA	NA
5	2020 Jan 23	2020 Jan 29	3,119	3,010	109	4	NA	NA
6	2020 Jan 30	2020 Feb 5	3,179	3,012	167	6	NA	NA
7	2020 Feb 6	2020 Feb 12	3,152	3,012	140	5	NA	NA
8	2020 Feb 13	2020 Feb 19	3,106	3,009	97	3	NA	NA
9	2020 Feb 20	2020 Feb 26	2,973	3,002	−29	−1	NA	NA
10	2020 Feb 27	2020 Mar 4	3,104	2,992	112	4	0	NA
11	2020 Mar 5	2020 Mar 11	3,081	2,980	101	3	7	14.5
12	2020 Mar 12	2020 Mar 18	3,286	2,964	322	11	99	3.3
13	2020 Mar 19	2020 Mar 25	3,941	2,946	995	34	502	2.0
14	2020 Mar 26	2020 Apr 1	4,764	2,926	1,838	63	1,003	1.8
15	2020 Apr 2	2020 Apr 8	5,143	2,903	2,240	77	1,144	2.0
16	2020 Apr 9	2020 Apr 15	4,565	2,879	1,686	59	941	1.8
17	2020 Apr 16	2020 Apr 22	4,109	2,853	1,256	44	837	1.5
18	2020 Apr 23	2020 Apr 29	3,692	2,827	865	31	558	1.5
19	2020 Apr 30	2020 May 6	3,153	2,801	352	13	365	1.0
20	2020 May 7	2020 May 13	2,876	2,774	102	4	240	0.4
21	2020 May 14	2020 May 20	2,788	2,748	40	1	136	0.3
22	2020 May 21	2020 May 27	2,694	2,723	NA	NA	112	−0.3
23	2020 May 28	2020 Jun 3	2,690	2,700	NA	NA	61	−0.2
24	2020 Jun 4	2020 Jun 10	NA	2,679	NA	NA	45	NA
25	2020 Jun 11	2020 Jun 17	NA	2,660	NA	NA	26	NA

*COVID-19, coronavirus disease; NA, not applicable. †Week as defined in the death surveillance (running Thursday through Wednesday).

### Death Monitoring

Once a week, the number of reported deaths is checked for excess above the number of expected deaths. For our analyses, we used deaths reported within 3 weeks (as were 99% of all deaths reported). Because data are received on Thursday morning, weekly numbers are aggregated from Thursdays through Wednesdays. A weekly email bulletin reporting the findings is sent to the Infectious Disease Early Warning Unit (at RIVM), and a short summary is placed weekly on the website (https://www.rivm.nl/monitoring-sterftecijfers-nederland). Any known concurrent and possibly related events are also reported. Data are sent weekly to EuroMOMO, (https://www.euromomo.eu), which monitors excess deaths at the level of Europe.

We used linear regression models to estimate current weekly baseline deaths on the basis of the preceding 5-year data wherein previous events were removed. Any deaths above the expected level was considered excess deaths and significantly increased when above the upper 95% prediction limit. In addition, a range of excess deaths was provided by calculating excess deaths as observed deaths minus the upper limit and observed deaths minus the lower limit. We provide further details of the statistical model and additional calculations (Appendix). 

## Results

The average weekly number of deaths was 2,797 during 2010–2020. Death numbers were generally higher in winter than in summer months and showed an upward trend over time, related to an aging population.

### Excess Deaths

**Figure 1:**
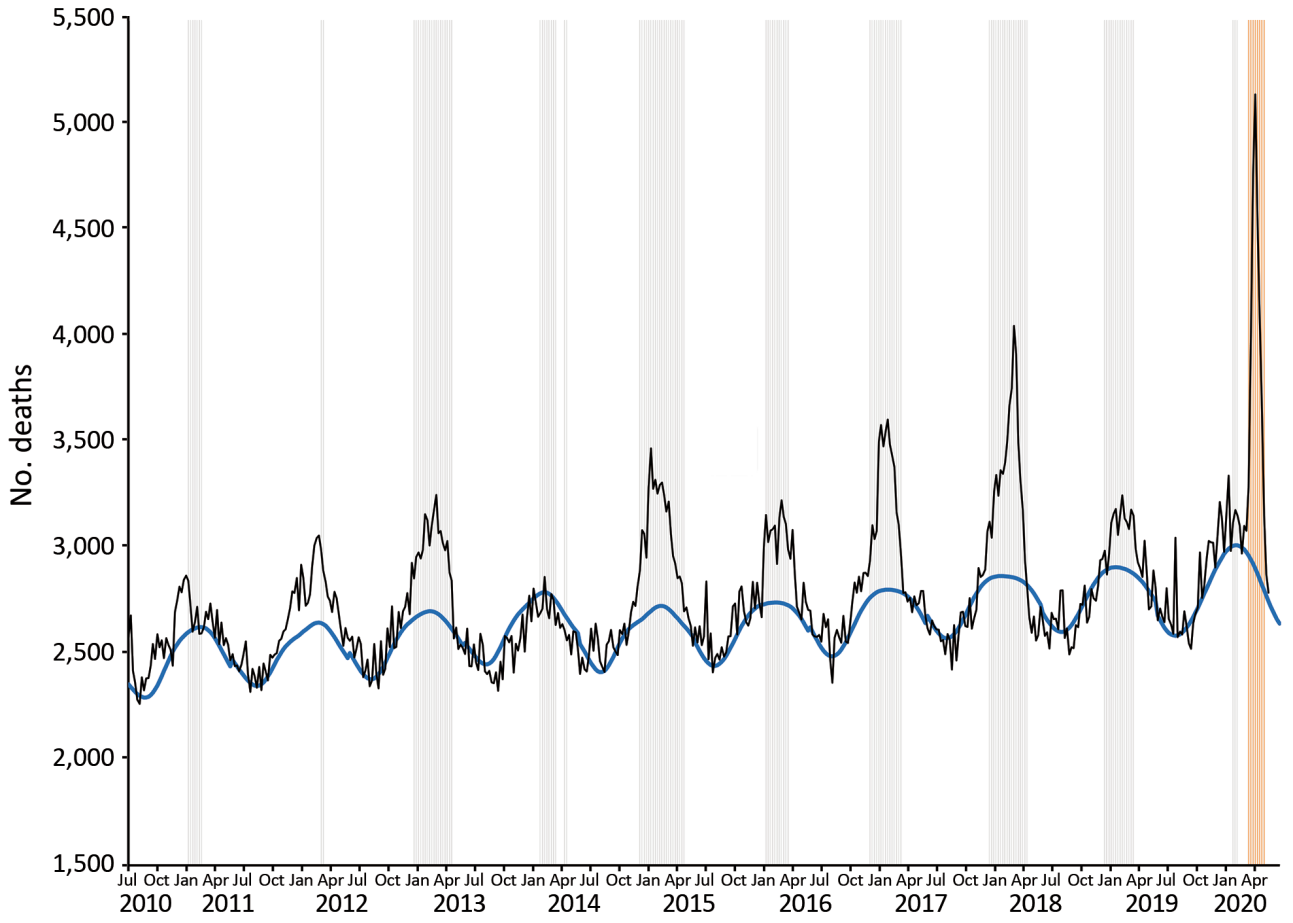
Excess deaths during influenza and coronavirus disease (COVID-19) and infection-fatality rate for severe acute respiratory syndrome coronavirus 2, the Netherlands. Weekly and expected number of deaths, 2010–2020. Black line indicates weekly number of deaths, and blue line indicates expected number of weekly deaths. Blue vertical bars indicate influenza epidemic weeks, and orange vertical bar indicates COVID-19 epidemic week 12–19 (March 12–May 6); excluding week 10–11, which overlapped with an influenza epidemic flare-up. Weeks run Thursday through Wednesday.

Using our death algorithm, we found that excess deaths were found during all previous influenza epidemics except that during 2013–2014 (Figure 1). For influenza, deaths reached their highest weekly peak during the 2017–2018 epidemic when 4,049 deaths were observed in week 10, and 2,860 deaths was the expected baseline (deaths reported within 3 weeks).

**Figure 2:**
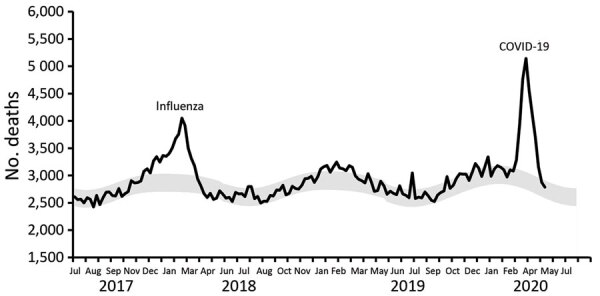
Excess deaths during influenza and COVID-19 and infection-fatality rate for severe acute respiratory syndrome coronavirus 2, the Netherlands. Weekly deaths and expected baseline deaths (July 2017–June 2020). Black line indicates weekly number of deaths (black line). Weeks run Thursday through Wednesday. Gray shading indicates lower and upper limits of expected baseline weekly deaths. COVID-19, coronavirus disease.

Excess deaths were found during the COVID-19 epidemic (weeks 12–19 2020; Thursday, March 12, through Wednesday, May 6) and reached its peak in week 15 (April 2–8) with 5,143 deaths; 2,903 was the expected baseline level (thus 77% in excess) for deaths reported within 3 weeks (Figure 2). COVID-19 excess deaths peaked 5–6 weeks after the first COVID-19 patient was detected on February 27. By week 20, deaths were not greatly increased beyond expected levels, but reports of COVID-19 deaths continued at low levels.

No COVID-19 deaths were reported in week 10 (February 27–March 4); 7 were reported in week 11 (March 5–11), the first 2 COVID-19 weeks with a concurrent influenza epidemic. Reported COVID-19 deaths increased to 99 in the ensuing week 12 (March 12–18) by which time the influenza epidemic had receded. Reported COVID-19 deaths peaked at 1,144 in week 15 (April 2–8), coinciding with peak excess deaths.

### Ratio of Excess Deaths to COVID-19 Deaths

**Figure 3:**
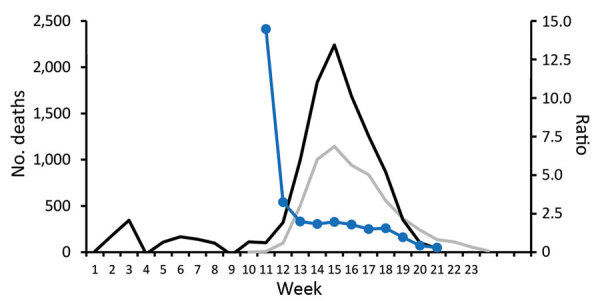
Excess deaths during influenza and coronavirus disease (COVID-19) and infection-fatality rate for severe acute respiratory syndrome coronavirus 2, the Netherlands. Excess deaths, reported COVID-19 deaths, and ratio between the 2 items (weeks 1–25, 2020). Black lines indicates excess deaths, gray line indicates COVID-19 reported deaths, and blue line indicates the ratio. Excess deaths were estimated with deaths reported within 3 weeks (and thus not yet available for week 24–25). The ratio for week 11 was 14.5, during the second (and final) week of the mixed influenza and COVID-19 epidemic.

Excess deaths were 3.3 times higher than reported COVID-19 deaths in week 12 (March 12–18) but then stabilized at 1.8–2.0 times higher in the ensuing 4 weeks (weeks 13–16; ratio 2.0 during peak deaths in week 15) (Figure 3). The ratio then further decreased to 1.5 in weeks 17 and 18 (April 16–29) and had decreased to 1 in week 19 (April 30–May 6), the final week with excess deaths for our model.

### Accumulated Excess Deaths

On average, ≈4,000 accumulated excess deaths were observed during influenza epidemics, but numbers varied considerably from 1 epidemic to another (Table 2). During the 2013–14 influenza epidemic, observed deaths were around baseline levels. During the other influenza epidemics, excess deaths varied from the lowest estimates of 404–600 (2010–2011, 2011–2012, and 2019–2020) up to 9,373 (range 6,439–12,306) during 2017–2018. During the 2017–18 epidemic, the accumulated all-cause deaths were 60,790, or 18% higher than the expected baseline level of 51,417. Influenza epidemic duration varied from 2 to 21 weeks; longer epidemics tended to show higher excess deaths.

**Table 2:** Excess deaths during influenza epidemics and the COVID-19 epidemic, the Netherlands*

Season	Influenza strains†	Influenza vaccine match‡	Vaccinated, % of target group§	Epidemic duration, weeks	Excess deaths	
No. (range)¶	% Above expected deaths
2010–2011	A(H1N1)pdm09 dominance followed by B Victoria dominance	Match	69	7	416 (−722 to 1,555)	2
2011–2012	A(H3N2) dominance	Mismatch	66	2	600 (308–892)	11
2012–2013	Mixed A(H1N1)pdm09 and A(H3N2) dominance followed by mixed B Yamagata and Victoria dominance	Mismatch	62	18	6,318 (3,790–8,846)	13
2013–2014	Mixed dominance with slightly more A(H3N2) than A(H1N1)pdm09	Mismatch	60	11	−581 (−1,927 to –765)	−2.1
2014–2015	A(H3N2) dominance followed by B Yamagata dominance	Mismatch	57	21	8,574 (5,831–11,316)	15
2015–2016	A(H1N1)pdm09 dominance followed by B Victoria dominance	Match	56	11	3,883 (2,390–5, 375)	13
2016–2017	A(H3N2)	Match	54	15	7,527 (5,236–9,817)	18
2017–2018	B Yamagata dominance; at end of season mixed AH3N2 and A(H1N1)pdm09 dominance	Mismatch; match#	50	18	9,373 (6,439–12,306)	18
2018–2019	Mixed A(H1N1)pdm09 and AH3N2 dominance	Match	51	14	2,858 (499–5,217)	7
2019–2020	Mixed A(H1N1)pdm09 and AH3N2 dominance	Match	53	3**	404 (−97 to –905)	4
2019–2020	Mixed Influenza and COVID-19 epidemic (weeks 10 and 11, 2020)	Match	NA	2	213 (−115 to −541)	4
2019–2020	COVID-19 (selected weeks: 12–19, 2020)	NA	NA	8††	9,554 (8,271–10,838)	41
2010–2020	Influenza seasonal average	NA	NA	12	3,995	10

*COVID-19, coronavirus disease; NA, not applicable; pdm, pandemic. †COVID-19 or influenza epidemic (influenza strains as reported in the Annual report: surveillance of influenza and other respiratory infections in the Netherlands). ‡Vaccine match with the dominant influenza strain(s). §Persons >60 years of age or with concurrent conditions and increased risk for influenza complications, % as reported previously (*16**–**19*). ¶Number of deaths above expected baseline number of deaths (observed minus expected deaths). The range is approximated by the lower limit minus observed and the upper limit minus observed. #Mismatch: Yamagata was not included in the vaccine; match: for both influenza A H1 and H3 strains.    **Excluding weeks 10 and 11 in 2020, which were both COVID-19 and influenza epidemic weeks (COVID-19: week 12–19: Thursday March 12–Wednesday May 6).  ††Weeks with high excess deaths in the first wave of the COVID-19 epidemic.

The total number of excess deaths during weeks 12–19 (March 12–May 6) of the COVID-19 epidemic was estimated to be 9,554 (range 8,271–10,838) (Table 2), which is almost twice (1.8 times) the total of 5,449 reported COVID-19 deaths during the same period. A total of 32,654 accumulated all-cause deaths were observed during this period, whereas accumulated expected deaths were 23,099 (deaths reported within 3 weeks). Thus, observed deaths were an overall 41% higher than expected baseline levels. In the 2 weeks that had a mixed influenza and COVID-19 epidemics (weeks 10 and 11; February 27–March 11, 2020), excess deaths totaled 213 (range –115 to 541).

### Estimated SARS-CoV-2 Infection-Fatality Rate

The assumption that all 9,554 excess deaths were associated directly with COVID-19 infections is an oversimplification, but enables a provisional estimate of the infection-fatality rate. In April 2020, the first 2 national serologic surveys by Sanquin (*20*) and Pienter (*21*) provided provisional estimates of the proportion of persons who had SARS-CoV-2 antibodies: 3% of adult blood donors (*20*) and 4% of a random population sample (*21*). A second blood donor survey reported a subsequent preliminary estimate of 5.5% on the basis of blood samples drawn May 10–20, 2020 (*22*). This finding suggests that up to 5.5% of the general population had experienced a SARS-CoV-2 infection by early May 2020. Of the total population size of 17.3 million (Statistics Netherlands, 2019), 5.5% corresponds to an estimated 951,500 coronavirus-infected persons (0.055 × 17.3 million = 951,500), thus placing the preliminary estimated overall infection-fatality rate at 1% (9,554 excess deaths/951,500 infected persons).

### Estimated Potential COVID-19 Deaths without Control Measures

A series of hygiene, social-distancing and partial lockdown measures have been implemented since March 9, 2020 (week 11; March 5–11 for our data). These measures and dates include cease handshaking, March 9; work from home, March 12; closure of schools and bars/restaurants, March 15; and stay-at-home advised and contact professions banned, March 23. During the COVID-19 epidemic, the RIVM provided weekly analyses and forecasts of the epidemic in the form of estimates of the virus reproduction number and projections of intensive care admissions by using a dynamic transmission model fitted to intensive care data (*23*). On the basis of the estimated reproduction number before the start of control measures (≈2–2.5), and model simulations in absence of control, it is expected that the epidemic would have infected 75%–80% of the population by early June 2020. This range is ≈14 times the 5.5% (*22*) seroprevalence found in May 2020. Assuming the same estimated infection-fatality rate, this rate would have resulted in 9,554 × 14, or 134,000, excess deaths if no control measures had been in place. This value is 0.78% of the population of the Netherlands.

## Discussion

Excess deaths varied considerably among influenza epidemics; the highest level in the past 10 years was observed during the influenza epidemic of 2017–18; there were an estimated 9,373 excess deaths in 18 weeks. The excess deaths for COVID-19 was similar in number to this large influenza epidemic, but its 9,554 excess deaths occurred in a much shorter time period (8 weeks) and reached a higher weekly peak (5,143) than during the influenza epidemic (4,049). In addition, the measures implemented to control the COVID-19 epidemic presumably prevented many infections (*24*) and deaths. Thus, the effect of COVID-19 on deaths is potentially much higher than that of seasonal influenza. The joint effect of influenza and COVID-19 epidemics on deaths is not yet known because they hardly overlapped during the past influenza season. To avoid miscomparisons (*25*), we compared excess deaths from influenza and COVID-19 by using the same data (all-cause deaths) and the same statistical method.

The case-fatality rate (CFR), calculated as the proportion of laboratory-confirmed COVID-19 cases that are fatal, is a commonly used measure of the severity of the COVID-19 epidemic. However, the CFR is greatly affected by testing and reporting practices and therefore cannot be used for comparisons over time and among countries. Some countries report only laboratory-confirmed cases, whereas other countries report clinically suspected patients and deaths. In addition, countries that test persons who have mild symptoms will have lower CFRs than countries that restrict testing to severely ill persons. We therefore calculated the infection-fatality rate on the basis of excess deaths and results of seroepidemiologic studies, a measure suitable for international comparisons.

The serologic surveys from which we used the estimated proportion of the population infected with SARS-CoV-2 are still in progress, and follow-up results will help to further improve the infection-fatality rate estimate. Cross-reactivity of laboratory tests might have caused an underestimation of the infection-fatality rate, whereas delayed antibody production after infection might have caused an overestimation. Additional study is required to better estimate the number of COVID-19 deaths averted in the Netherlands.

We used a ratio of ≈14-fold higher expected excess deaths (134,000, or 0.78% of the population) in the absence of mitigation measures. This ratio is crude and not published but aligns with estimates from 11 other countries in Europe, which report COVID-19 deaths ranging from 0.22% to 1.1% of the population had there been no interventions (*26*). Without mitigation measures, persons might also have changed behavior, which would have affected the currently assumed ratio of 14.

The death surveillance system in the Netherlands was set up during the 2009 influenza pandemic to track the effect and burden of any epidemic and to signal any unexpected or undetected events (*1*,*8*,*9*). All calculations of excess deaths in this study and in our death surveillance are estimations and thus provide only a preliminary estimate of excess deaths from COVID-19. The straightforward linear regression model with linear and harmonic terms assumes a normal error distribution with constant variance, an approximation we deemed applicable to high numbers of weekly deaths.

There is no standard for determining actual expected levels of deaths and various calculations exist, even within the same country (*27*). Our method is similar to the regression method used by the EuroMOMO network (*28**,**29*) and similar to Serfling-type regression models (i.e., including seasonality by using sine and cosine terms) (*30*–*32*). However, the true baseline level of deaths during winter in the presence of influenza epidemics remains difficult to estimate. Removing seasons (*28*) or extremes to estimate the baseline warrants additional future sensitivity analyses. Our model detects no excess deaths in 2013–14, corresponding to a previous estimate of no influenza-associated intensive care admissions in that season (*33*). By accumulating the difference between the observed number of deaths and the upper (or lower) limit of the predicted baseline number of deaths, we only approximated the 95% prediction intervals. The intervals obtained in this way are too wide because nonlinearities in the calculation are neglected. Instead, in the future, by applying Monte Carlo simulation, we could obtain a better approximation of the 95% prediction intervals.

Weekly excess deaths from COVID-19 were usually 1.5–2-fold higher than those reported, indicating the extent of potential underreporting or underdetection of COVID-19 deaths. This discrepancy was greater at the beginning of excess deaths (at the peak of the COVID-19 epidemic), most likely because many regional public health services were unable to follow all the reported patients for disease outcome, including death status. All excess deaths during weeks 12–19 were most likely caused by direct and indirect effects of SARS-CoV-2 infections. No other outbreaks or extreme weather events were present during the epidemic weeks. Reported circulation of many other infectious diseases was actually lower than expected (*34*), probably because of partial lockdown, social distancing, and hygiene instructions.

We currently do not know the distribution of direct versus indirect effects of the COVID-19 epidemic on deaths. A major indirect factor to be explored is the postponement of regular medical care, especially during the peak of the epidemic. Hospitals were overburdened and halted admissions for nonurgent care. Also, healthcare-seeking behavior changed in patients with nonrespiratory symptoms because they feared getting COVID-19 in hospitals or putting additional pressure on the healthcare system. Other indirect effects on deaths might have been caused by shifts (up or down) in occurrence of potentially fatal events, such as accidents and suicides. In-depth analyses of death-cause data will shed more light on these events.

Several other issues should also be elucidated in further studies. First, we provided an indication of excess deaths in the total population of the Netherlands, but it occurred mostly, but not exclusively, in the elderly (groups >65 years of age) during the influenza epidemics and the COVID-19 epidemic. Age-specific results warrant further investigation and reporting, as do regional differences (*35*). Second, our analyses provide estimates specific to the Netherlands. Data for the Netherlands are also included in the EuroMOMO death monitoring, which pools data from 24 countries and provides death surveillance at a level for Europe. Third, influenza epidemics, which are well monitored, are the most frequent infectious disease events coinciding with excess deaths in the Netherlands. However, influenza epidemics often coincide with other respiratory infections in winter or (occasionally) cold weather. Influenza is a well-known contributor to excess deaths, but our methods did not disentangle its contribution from that of other respiratory infections and events. Fourth, the estimate of COVID-19 excess deaths was based on data for weeks 12–19, after which overall death rates returned to baseline levels. However, COVID-19 deaths were still reported at low levels (e.g., 45 during week 24), and we do not know how COVID-19 deaths will continue to evolve.

Although COVID-19 incidence has greatly decreased because of social distancing and lockdown measures, measures are still in place to reduce virus transmission. The 9,554 excess deaths (March 12–May 6) are a slight underestimation of the total excess during the entire COVID-19 epidemic because we excluded the first 2 weeks in which influenza and COVID-19 epidemics coincided. With excess deaths at 213 during those weeks, this exclusion underestimates COVID-19 excess deaths by at most 2%. An additional 1% underestimation is caused by using deaths reported within 3 weeks (i.e., 99% of deaths reported), which is an input parameter for the weekly algorithm in death monitoring. Finally, further quantification of years of life lost because of COVID-19 is required because such loss may be considerable (*36*).

Influenza vaccination is available in the Netherlands for risk groups (persons >60 years of age or those with underlying conditions) to reduce severe sequelae of influenza infections, but coverage is rather low (51% in 2019) (*33*–*36*). Vaccination is only partially effective, and the effectiveness varies by season because of virus strain variability and varying vaccine match (Table 2). COVID-19 vaccination is not yet available. Social distancing and lockdown measures have had a large effect on decreasing the epidemic and thus also COVID-19 deaths. If some or all of these measures stay in place, they might likewise decrease influenza virus circulation and thus severe sequelae of infection in the upcoming winter season, as observed in Hong Kong, China, at the beginning of the COVID-19 epidemic (*37*).

The 2019–2020 influenza season just preceding the COVID-19 epidemic was short and relatively mild; there were 404 excess deaths compared with an average of 4,000 in seasons over the past 10 years. It is unknown whether excess deaths would have differed had the COVID-19 epidemic been preceded by a more severe influenza epidemic or a colder winter. It is likewise unclear how SARS-CoV-2 and influenza virus infections might interact and affect deaths, should epidemics occur simultaneously. In our study, the short mixed-epidemic period of 2 weeks did not involve full combined virus circulation because the influenza epidemic was decreasing and the COVID-19 epidemic was just getting started.

In conclusion, estimation of excess deaths complements the reporting of laboratory-confirmed COVID-19 deaths, indicating the potential magnitude of underreporting and underdetection of COVID-19 deaths. These estimates also provide a timely indication of the combined direct and indirect effects of the COVID-19 epidemic on population deaths. In the coming weeks and months, monitoring of deaths remains key to the timely monitoring of the effects of COVID-19 and influenza. COVID-19 might have a long-lasting effect, potentially becoming endemic with yearly recurrence(s), similar to influenza. It remains to be seen whether the effect of COVID-19 on deaths remains greater than that of influenza. Monitoring of excess deaths can provide input for public health and economic decisions. This monitoring also remains essential for monitoring the effects of any other events and outbreaks and for detecting any unexpected and unforeseen increases in deaths.

Appendix Additional information on excess deaths during influenza and coronavirus disease and infection-fatality rate for severe acute respiratory syndrome coronavirus 2, the Netherlands.
