# Supplementary material for: Excess Deaths during Influenza and Coronavirus Disease and Infection-Fatality Rate for Severe Acute Respiratory Syndrome Coronavirus 2, the Netherlands
Source: Emerg Infect Dis. 2021 Feb;27(2):411–20. doi: 10.3201/eid2702.202999 (PMC7853586; doi:10.3201/eid2702.202999)
Supplement: Appendix — Additional information on excess deaths during influenza and coronavirus disease and infection-fatality rate for severe acute respiratory syndrome coronavirus 2, the Netherlands. [file 20-2999-Techapp-s1.pdf]

Article DOI: https://doi.org/10.3201/eid2702.202999

Excess Deaths during Influenza and Coronavirus Disease and Infection-Fatality Rate for Severe Acute Respiratory Syndrome Coronavirus 2, the Netherlands

Appendix

Statistical Methods

For each year, we estimated the weekly baseline death level M_w based on the previous 5-year data by using a linear regression with a linear time trend and up to 3 sine and cosine terms to capture cyclical seasonal trends:

$$M_w = \beta_0 + \beta_1 w + \sum_{k=1}^{3}\left[\beta_{2,k}\sin\left(\frac{2\pi kw}{52.18}\right) + \beta_{3,k}\cos\left(\frac{2\pi kw}{52.18}\right)\right] + \epsilon_w$$

where w indicates the week index, running from 1 to 260 or 261 weeks, depending on the number of weeks in the 5-year period. The different values for k lead to cycles of 1 year and 6 and 4 months to ensure sufficient flexibility. The βs are unknown regression coefficients and ϵ_w is the residual error term, which is assumed to be normal with constant variance. Only significant terms ($p<0.05$) are included in the baseline models by applying backward elimination.

Before fitting the baseline models, weeks with underreported deaths were removed; they often coincided with public holidays. The 7.5% most underreported weeks were removed (e.g., for the 2020 baseline, these were weeks wherein <95.5% of total deaths were registered within 3 weeks of death). Also, periods with high excess deaths in winter (25% of highest observations) and summer (20% of the highest observations in July and August) were removed so as not to influence the baseline with previous events. Fall and spring values contributed most to baseline determination, similarly to other studies (*1*,*2*).

We assume the baseline to represent expected death levels not impacted by previous events, such as outbreaks (e.g., influenza epidemics and other respiratory infections in winter) and extreme temperatures (e.g., heat waves and extreme cold). Baselines are updated each year in June. The upper and lower limits of the baseline are based on the 95% prediction intervals, representing the range wherein the baseline deaths fluctuate. Any deaths above the expected level is considered excess deaths

(i.e., observed deaths minus the center of the baseline prediction) and is statistically significantly increased when above the upper 95% prediction limit. In addition, a range of excess deaths was provided by calculating excess deaths as observed deaths minus the upper limit and observed deaths minus the lower limit.

Cumulative excess deaths across weeks provided total excess deaths during each influenza epidemic in 2010–2020; during 8 weeks of the coronavirus disease (COVID-19) epidemic in weeks 12–19 (March 12–May 6); and during 2 weeks of the mixed influenza–COVID-19 epidemic in weeks 10–11 (February 27–March 11). The infection-fatality rate of COVID-19 was calculated as excess deaths divided by estimated total number of persons infected with coronavirus by early May. This number of infected persons was estimated as the seropositive proportion (determined by a national survey) multiplied by the total population size of the Netherlands.

References

1. Molbak K, Espenhain L, Nielsen J, Tersago K, Bossuyt N, Denissov G, et al. Excess mortality among the elderly in European countries, December 2014 to February 2015. Euro Surveill. 2015;20:21065. PubMed https://doi.org/10.2807/1560-7917.ES2015.20.11.21065

2. Baghdadi Y, Gallay A, Caserio-Schönemann C, Fouillet A. Evaluation of the French reactive mortality surveillance system supporting decision making. Eur J Public Health. 2019;29:601–7. PubMed https://doi.org/10.1093/eurpub/cky251
